# Real-World Outcomes and Progression Patterns with First-Line Osimertinib in Patients with Advanced *EGFR*-Mutant Non-Small Cell Lung Cancer: A Nationwide Turkish Oncology Group Study

**DOI:** 10.3390/cancers18121979

**Published:** 2026-06-18

**Authors:** Bahadır Köylü, Özde Melisa Celayir, Harun Muğlu, Orçun Can, Fatma Şen, Bala Başak Öven, Umut Demirci, Teoman Şakalar, Serdar Karakaya, Özlem Er, Ömer Burak Ekinci, Abdullah Sakin, Hasibe Bilge Gür, Yağmur Karaman, Duygu Ercan Uzundal, Esra Aşık, Kezban Nur Pilancı, Nilüfer Avcı, Pınar Gürsoy, Muhammet Ali Kaplan, Serkan Menekşe, Engin Kut, Erkan Arpacı, Hakan Kosku, Selami Bayram, Taha Koray Şahin, Emine Türkmen, Abdurrahman Aykut, Yeşim Ağyol, Ahmet Özveren, Elif Şahin, Öztürk Ateş, Gamze Serin Özel, Cevat İlteriş Kıkılı, Fatih Kemik, Başak Oyan, Taner Korkmaz, Osman Gökhan Demir, Deniz Tural, Şeyda Gündüz, Şahin Laçin, Nazan Demir, Didem Tunalı, Ahmet Bilici, Ömer Fatih Ölmez, İlhan Hacıbekiroğlu, Erdem Çubukçu, Ozan Yazıcı, Atila Yıldırım, Burak Bilgin, Mehmet Ali Nahit Şendur, Ali Murat Tatlı, Mustafa Özdoğan, Burak Yasin Aktaş, Metin Kanıtez, Bülent Karabulut, Ferhat Ekinci, Ertuğrul Bayram, Gamze Gököz Doğu, Saadettin Kılıçkap, Perran Fulden Yumuk, Nil Molinas Mandel, Fatih Selçukbiricik

**Affiliations:** 1 Division of Medical Oncology, Department of Internal Medicine, Koç University School of Medicine, Istanbul 34010, Türkiye; ikikili@ku.edu.tr (C.İ.K.); fkemik@ku.edu.tr (F.K.); salacin@kuh.ku.edu.tr (Ş.L.); fuldeny@amerikanhastanesi.org (P.F.Y.); nilm@amerikanhastanesi.org (N.M.M.); fselcukbiricik@ku.edu.tr (F.S.); 2 Department of Medical Oncology, Başakşehir Çam and Sakura City Hospital, Istanbul 34480, Türkiye; ozdemelisa.celayir@sbu.edu.tr; 3 Division of Medical Oncology, Department of Internal Medicine, Medipol University School of Medicine, Istanbul 34214, Türkiye; hm1635@hotmail.com (H.M.); abdullah.sakin@medipol.edu.tr (A.S.); abilici@medipol.edu.tr (A.B.); 4 Division of Medical Oncology, Department of Internal Medicine, Acibadem University, Istanbul 34752, Türkiye; orcun.can@acibadem.com (O.C.); ozlem.er@acibadem.com (Ö.E.); basak.uluc@acibadem.com (B.O.); taner.korkmaz@acibadem.com (T.K.); gokhan.demir@acibadem.com (O.G.D.); 5 Division of Medical Oncology, Department of Internal Medicine, Uskudar University Faculty of Medicine, Istanbul 34768, Türkiye; fatma.sen@uskudar.edu.tr; 6 Department of Medical Oncology, Yeditepe University, Istanbul 34755, Türkiye; basak.oven@yeditepe.edu.tr; 7 Department of Medical Oncology, Memorial Ankara Hospital, Ankara 06520, Türkiye; umut.demirci@memorial.com.tr; 8 Department of Medical Oncology, HG Hospital, Kahramanmaras 46050, Türkiye; drteomansakalar@gmail.com; 9 Department of Medical Oncology, Ankara Atatürk Sanatorium Training and Research Hospital, Ankara 06290, Türkiye; drserdarkarakaya@gmail.com; 10 Department of Medical Oncology, Prof. Dr. Cemil Tascioglu City Hospital, Istanbul 34384, Türkiye; omerburak.ekinci@saglik.gov.tr; 11 Division of Medical Oncology, Department of Internal Medicine, Sakarya University Faculty of Medicine, Sakarya 54050, Türkiye; hgur@sakarya.edu.tr (H.B.G.); ihacibekiroglu@sakarya.edu.tr (İ.H.); 12 Division of Medical Oncology, Department of Internal Medicine, Uludag University Faculty of Medicine, Bursa 16059, Türkiye; yagmurkaraman@uludag.edu.tr (Y.K.); erdemcubukcu@uludag.edu.tr (E.Ç.); 13 Division of Medical Oncology, Department of Internal Medicine, Gazi University, Ankara 06560, Türkiye; duygu.ercanuzundal@saglik.gov.tr (D.E.U.); ozanyazici@gazi.edu.tr (O.Y.); 14 Division of Medical Oncology, Department of Internal Medicine, Karadeniz Technical University Faculty of Medicine, Trabzon 61080, Türkiye; dr.esrakaya@ktu.edu.tr (E.A.); atilayildirim@ktu.edu.tr (A.Y.); 15 Department of Medical Oncology, Bahçelievler Memorial Hospital, Istanbul 34180, Türkiye; kezban.pilanci@memorial.com.tr; 16 Radiotherapy Programme, Vocational School of Health Services, Fenerbahce University, Istanbul 34758, Türkiye; nilufer.avci@fbu.edu.tr; 17 Division of Medical Oncology, Department of Internal Medicine, Ege University Faculty of Medicine, Izmir 35040, Türkiye; pinar.gursoy@ege.edu.tr; 18 Division of Medical Oncology, Department of Internal Medicine, Dicle University Faculty of Medicine, Diyarbakır 21280, Türkiye; makaplan@dicle.edu.tr; 19 Department of Medical Oncology, Manisa City Hospital, Manisa 45040, Türkiye; drsermen@hotmail.com (S.M.); engin.kut@saglik.gov.tr (E.K.); 20 Department of Medical Oncology, Adatıp Hospital, Sakarya 54050, Türkiye; earpaci@adatiphastanesi.com; 21 Department of Medical Oncology, Ankara Bilkent City Hospital, Ankara 06800, Türkiye; hakankosku@gmail.com; 22 Department of Medical Oncology, Antalya Memorial Hospital, Antalya 07025, Türkiye; selami.bayram@memorial.com.tr (S.B.); murat.tatli@memorial.com.tr (A.M.T.); mustafa.ozdogan@memorial.com.tr (M.Ö.); 23 Department of Medical Oncology, Hacettepe University Cancer Institute, Ankara 06230, Türkiye; koraysahin@hacettepe.edu.tr (T.K.Ş.); byaktas@hacettepe.edu.tr (B.Y.A.); 24 Division of Medical Oncology, Department of Internal Medicine, Manisa Celal Bayar University Faculty of Medicine, Manisa 45140, Türkiye; emine.turkmen@cbu.edu.tr (E.T.); ekinci.ferhat@cbu.edu.tr (F.E.); 25 Division of Medical Oncology, Department of Internal Medicine, Çukurova University Faculty of Medicine, Adana 01330, Türkiye; aaykut@cu.edu.tr (A.A.); ertugrulbayram@cu.edu.tr (E.B.); 26 Division of Medical Oncology, Department of Internal Medicine, Marmara University Faculty of Medicine, Istanbul 34899, Türkiye; yesim.agyol@marmara.edu.tr; 27 Department of Medical Oncology, Acibadem Izmir Kent Hospital, Izmir 35620, Türkiye; ahmet.ozveren@acibadem.com (A.Ö.); bulent.karabulut@acibadem.com (B.K.); 28 Department of Medical Oncology, Kocaeli City Hospital, Kocaeli 41060, Türkiye; elif.sahin28@saglik.gov.tr; 29 Department of Medical Oncology, Dr. Abdurrahman Yurtaslan Ankara Oncology Training and Research Hospital, Ankara 06200, Türkiye; ozturk.ates@sbu.edu.tr; 30 Division of Medical Oncology, Department of Internal Medicine, Pamukkale University Faculty of Medicine, Denizli 20160, Türkiye; gozel@pau.edu.tr (G.S.Ö.); ggokoz@pau.edu.tr (G.G.D.); 31 Department of Medical Oncology, Koç University Hospital, Istanbul 34010, Türkiye; dtural@kuh.ku.edu.tr (D.T.); segunduz@kuh.ku.edu.tr (Ş.G.); ndemir@kuh.ku.edu.tr (N.D.); dtunali@kuh.ku.edu.tr (D.T.); 32 Department of Medical Oncology, Acibadem Maslak Hospital, Istanbul 34457, Türkiye; ofolmez@acibadem.com; 33 Division of Medical Oncology, Department of Internal Medicine, Ankara Yildirim Beyazit University Faculty of Medicine, Ankara 06800, Türkiye; burakbilgin@aybu.edu.tr (B.B.); masendur@yahoo.com.tr (M.A.N.Ş.); 34 Department of Medical Oncology, American Hospital, Istanbul 34365, Türkiye; metinka@amerikanhastanesi.org; 35 Division of Medical Oncology, Department of Internal Medicine, Istinye University Faculty of Medicine, Istanbul 34010, Türkiye; saadettin.kilickap@istinye.edu.tr

**Keywords:** epidermal growth factor receptor, tyrosine kinase inhibitor, lung neoplasms, radiotherapy

## Abstract

**Background/Objectives**: We aimed to evaluate the real-world outcomes of first-line osimertinib in patients with advanced *EGFR*-mutant non-small cell lung cancer, together with the progression patterns, including the management and outcomes of oligoprogressive disease in the real-world setting. **Methods**: Patients who received first-line osimertinib at 33 oncology centers across Türkiye were retrospectively analyzed. **Results**: The median (IQR) age of 143 patients was 59.4 (50.9–68.9), 62.9% were female, and 68.5% were non-smokers. The most frequently identified *EGFR* mutations were exon 19 deletion (64.3%) and exon 21 L858R (28.7%). The median progression-free survival (PFS) was 17.6 months (95% CI 14.6–20.6). Exon 19 deletion subgroup had significantly longer median PFS than exon 21 L858R subgroup (22.0 vs. 11.7 months; HR = 0.40 [95% CI 0.25–0.64]; *p* < 0.001). Of the patients who experienced disease progression, 51.2% had oligoprogression. Among these, 81.8% received local ablative treatments (LATs) while continuing osimertinib. The median time to osimertinib discontinuation was significantly longer in patients treated with LATs than in those who continued osimertinib without LATs (8.5 vs. 3.0 months; *p* = 0.001). In the overall cohort, 12- and 24-month overall survival (OS) rates were 93.0% and 82.5%, respectively. The median OS was significantly longer in patients with oligoprogression compared to those with systemic progressive disease (57.7 vs. 36.5 months; *p* = 0.007). Any-grade and grade ≥ 3 osimertinib-related adverse events occurred in 55.2% and 6.3% of patients, respectively. **Conclusions**: This study supports the real-world effectiveness and tolerability of first-line osimertinib. Oligoprogression was associated with longer OS compared with systemic progression, and the integration of LATs in carefully selected patients with oligoprogression may be associated with prolonged osimertinib treatment duration and favorable post-progression outcomes.

## Simple Summary

Epidermal growth factor receptor (*EGFR*) mutations represent the second most common oncogenic driver in non-small cell lung cancer. Osimertinib, a third-generation *EGFR*-tyrosine kinase inhibitor, has demonstrated improved survival outcomes compared with first-generation *EGFR*-tyrosine kinase inhibitors. During osimertinib treatment, disease progression may occur with different patterns. In this nationwide, multicenter, retrospective study, we evaluated the real-world outcomes of first-line osimertinib in patients with advanced *EGFR*-mutant non-small cell lung cancer, together with the progression patterns. Oligoprogression rate was 51.2% and median overall survival was longer in case of oligoprogression than in systemic progression. While real-world median PFS mirrored that observed in pivotal trials, the integration of local ablative treatments in carefully selected patients with oligoprogression may be associated with prolonged osimertinib treatment duration and favorable post-progression outcomes. The intracranial efficacy outcomes were numerically similar to those reported in clinical trials.

## 1. Introduction

Epidermal growth factor receptor (*EGFR*) mutations represent the second most common oncogenic driver in non-small cell lung cancer (NSCLC), with a reported frequency of approximately 12–15% in European populations and 16.6% in Turkish population [1,2,3,4]. Activating *EGFR* mutations are more frequently identified among non-smokers, younger patients, and females [4]. Despite substantial advances in the systemic treatment of metastatic *EGFR*-mutant NSCLC, more than half of patients survive for less than four years [5].

Osimertinib, a third-generation *EGFR*-tyrosine kinase inhibitor (TKI), has demonstrated improved survival outcomes compared with first-generation *EGFR*-TKIs in the phase III FLAURA trial [6,7]. Recently, the addition of four cycles of platinum-based chemotherapy to osimertinib has been shown to improve overall survival (OS) in the phase III FLAURA2 trial, albeit at the cost of a higher incidence of grade 3 or higher adverse events [5]. However, many patients in real-world settings are unable to tolerate chemotherapy because of advanced age, organ dysfunction, or poor performance status. Additionally, while the combination of amivantamab and lazertinib showed superior progression-free survival (PFS) and OS compared with osimertinib monotherapy in the phase III MARIPOSA trial, the high rates of dermatologic toxicity, infusion-related events, and venous thromboembolism may limit its widespread use [8]. Therefore, osimertinib monotherapy continues to be widely used in clinical practice.

During osimertinib treatment, disease progression may occur with different patterns. Oligoprogressive disease is a relatively novel concept characterized by progression in a limited number of lesions (usually ≤3–5) with otherwise stable metastatic disease and is frequently observed in patients receiving osimertinib monotherapy, with reported rates ranging from 37% to 77% [9,10,11]. In cases of oligoprogression during osimertinib monotherapy, current guidelines support local ablative treatments with continuation of osimertinib, as this strategy may provide clinical benefit despite the lack of randomized phase III trials [12,13]. Substantial toxicities of subsequent-line systemic therapies and limited access to targeted agents, including amivantamab and lazertinib, further support the continuation of osimertinib in cases of oligoprogression in routine clinical practice.

In this study, we aimed to evaluate the real-world effectiveness and safety of first-line osimertinib monotherapy in patients with unresectable locally advanced or metastatic *EGFR*-mutant NSCLC. In addition, we explored patterns of disease progression, including the management and outcomes of oligoprogressive disease.

## 2. Materials and Methods

### 2.1. Study Design and Population

This nationwide, multicenter, retrospective study was conducted across 33 tertiary oncology centers in Türkiye. Patients with unresectable locally advanced or metastatic *EGFR*-mutant NSCLC who had not previously received systemic treatment for advanced disease and received first-line osimertinib monotherapy between June 2018 and December 2024 were retrospectively included. Inclusion criteria were as follows: age ≥ 18 years, histologically confirmed NSCLC with an *EGFR* mutation confirmed by a local laboratory, an Eastern Cooperative Oncology Group (ECOG) performance status of 0–2, and treatment with first-line osimertinib monotherapy. *EGFR* mutation status was determined at the participating centers using locally available validated molecular diagnostic methods. Next-generation sequencing was the most commonly used testing method, although PCR-based assays were also used in some centers according to local practice and test availability. Patients with missing essential clinicopathologic or survival follow-up data were excluded (Supplementary Figure S1). Baseline variables included demographics; clinicopathologic characteristics, including ECOG performance status, histologic subtypes, *EGFR* mutation type, PD-L1 expression, and metastatic sites at diagnosis; and treatment details, including local ablative treatments. Data were extracted from electronic medical records across participating centers using a standardized, prespecified data collection template.

### 2.2. Treatment Protocol and Follow-Up

Osimertinib was administered at a dose of 80 mg orally once daily as monotherapy and was continued until disease progression or unacceptable adverse events. Patients underwent regular follow-up according to local clinical practice at each participating center, with imaging assessments routinely performed every 8–12 weeks after initial evaluation. Baseline brain MRI was routinely performed as part of the initial staging evaluation. However, imaging modalities and the frequency of follow-up assessments, including central nervous system (CNS) imaging, were not fully standardized across participating centers and were determined at the discretion of the treating physicians. In cases of oligoprogression, defined as progression in ≤5 lesions during osimertinib treatment, osimertinib could be continued with or without local ablative treatments at the discretion of the treating physicians, in accordance with real-world clinical practice. CNS-only progression meeting this criterion was classified as oligoprogression, whereas leptomeningeal disease was considered systemic progression. For patients with oligoprogression, the number of local ablative treatments administered and the time to osimertinib discontinuation were also recorded.

### 2.3. Outcomes and Assessments

The primary endpoint of this study was to evaluate the effectiveness of osimertinib monotherapy in terms of PFS, defined as the time from initiation of osimertinib to disease progression or death, whichever occurred first. Secondary endpoints included OS, objective response rate (ORR), disease control rate (DCR), intracranial PFS and response rates, and osimertinib-related adverse events.

OS was defined as the time from initiation of osimertinib to death, with censoring at the last follow-up for patients who were alive or lost to follow-up at study end. Post-progression survival (PPS) was defined as the time from the first documented progression on osimertinib to death. Tumor responses were assessed by local investigators according to the Response Evaluation Criteria in Solid Tumors (RECIST), version 1.1 [14]. In accordance with real-world clinical practice, progression was also accepted when unequivocal radiologic progression was documented on PET-CT, particularly in the presence of new lesions or anatomically corresponding progressive disease confirmed by CT, MRI, or clinical evaluation. No centralized or blinded independent radiologic review was performed. Histopathological confirmation and/or additional confirmatory imaging were not mandatory for the diagnosis of progressive disease. ORR was defined as the proportion of patients achieving a complete response (CR) or partial response (PR), and DCR was defined as the proportion of patients with CR, PR, or stable disease (SD) as their best overall response according to RECIST version 1.1. Duration of response (DoR) was defined as the interval between the first documented objective response (CR or PR) and disease progression or death, whichever occurred first.

Safety was evaluated through the analysis of treatment-related adverse events (TRAEs), which were categorized and graded according to the National Cancer Institute Common Terminology Criteria for Adverse Events (CTCAE), version 5.0 [15].

### 2.4. Statistical Analysis

All statistical analyses were performed using SPSS software version 25.0 (IBM Corp., Armonk, NY, USA) and R version 4.5.1 (R Foundation for Statistical Computing, Vienna, Austria). No formal sample size calculation was performed due to the exploratory and descriptive nature of this retrospective study. The anticipated sample size was approximately 100 patients who met the predefined eligibility criteria.

Continuous variables were reported as medians with interquartile ranges (IQRs), whereas categorical variables were reported as frequencies and percentages. For descriptive analyses, missing values were reported as “Unknown” where applicable and are presented in the relevant tables. Analyses involving a specific variable were performed using available data for that variable. Kaplan–Meier methodology was used to estimate PFS, OS, PPS, DoR, and intracranial PFS, with comparisons between groups performed using the log-rank test. Follow-up duration was estimated using the reverse Kaplan–Meier method. Factors associated with PFS were evaluated using univariable and multivariable Cox regression analyses, with hazard ratios (HRs) and 95% confidence intervals (CIs) reported. Variables with *p* < 0.10 in univariable analysis were included in the multivariable model using the enter method. Given the limited number of death events, multivariable Cox regression analysis for OS was not performed to avoid model overfitting; OS was therefore assessed descriptively using Kaplan–Meier estimates and log-rank testing. A two-sided *p* value of <0.05 was considered statistically significant.

## 3. Results

A total of 159 patients treated with first-line osimertinib monotherapy between June 2018 and December 2024 were retrospectively enrolled in this nationwide multicenter study. Following the exclusion of 16 patients due to missing essential clinicopathologic or survival follow-up data, the final study population comprised 143 patients for efficacy and safety analyses (Supplementary Figure S1). Baseline clinicopathologic characteristics are presented for the overall study population and stratified according to the two most common *EGFR* mutations: exon 19 deletion and exon 21 L858R (Table 1). In the overall cohort, the median (IQR) age at diagnosis was 59.4 (50.9–68.9), and 90 (62.9%) patients were female. The majority of patients were non-smokers (*n* = 98; 68.5%) and had an ECOG performance status of <2 (*n* = 127; 88.8%). All patients had adenocarcinoma, with acinar (*n* = 27; 18.9%), solid (*n* = 25; 17.5%), and lepidic (*n* = 20; 14.0%) patterns being the most common predominant histologic subtypes. Patients with PD-L1 tumor proportion scores (TPS) of ≥1% and ≥50% represented 49.0% (*n* = 70) and 16.8% (*n* = 24) of the study population, respectively. The most frequently identified *EGFR* mutations were exon 19 deletion (*n* = 92; 64.3%) and exon 21 L858R (*n* = 41; 28.7%). In addition, rare *EGFR* mutations were observed in ten patients (7.0%), including de novo T790M (*n* = 5; 3.5%), exon 18 G719S, exon 18 G719X, exon 20 S768I, exon 20 in-frame insertion N771_P772insY, and exon 21 L861Q (each *n* = 1; 0.7%). Most patients presented with de novo metastatic disease (*n* = 124; 86.7%). The most frequent metastatic sites were bone (*n* = 76; 53.1%), CNS (*n* = 51; 35.7%), and contralateral lung (*n* = 32; 22.4%). The majority of patients had metastases in one or two organ systems (*n* = 118; 82.5%), while extensive metastatic disease involving ≥3 organ systems was present in 17.5% (*n* = 25) of the study population.

The median duration of follow-up was 23.1 months (95% CI 19.0–27.1). Following the diagnosis of metastatic disease, radiotherapy was administered to 24.5% of patients for CNS metastases and to 20.3% of patients for bone metastases. The median duration of osimertinib monotherapy was 13.7 months (95% CI 7.1–25.3). The ORR was 88.1% (95% CI 81.6–92.9%), and DCR was 93.0% (95% CI 87.5–96.6%) in the overall cohort (Supplementary Table S1). Patients in the exon 19 deletion subgroup achieved higher response rates, with an ORR of 92.4% and a DCR of 96.7%, compared with an ORR of 82.9% and a DCR of 87.8% in the exon 21 L858R subgroup.

**Figure 1:**
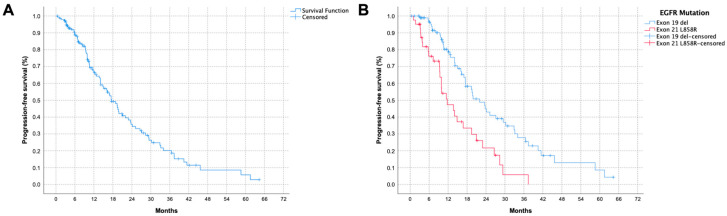
Kaplan–Meier curves for progression-free survival in (**A**) the overall cohort and (**B**) stratified by *EGFR* mutation subtype (exon 19 deletion vs. exon 21 L858R).

By the end of the data collection period, disease progression had occurred in 86 patients (60.1%). The median PFS in the overall cohort was 17.6 months (95% CI 14.6–20.6), with 12- and 24-month PFS rates of 67.3% (95% CI 59.3–76.4%) and 35.7% (95% CI 27.1–47.0%) (Figure 1). Compared with the exon 21 L858R subgroup, patients with exon 19 deletion had significantly longer median PFS (22.0 vs. 11.7 months; HR = 0.40 [95% CI 0.25–0.64]; *p* < 0.001), as well as higher 12-month (80.2% vs. 47.3%) and 24-month (44.9% vs. 21.7%) PFS rates. Of the patients who experienced progression on osimertinib monotherapy (*n* = 86), 44 (51.2%) had oligoprogression. The median DoR among patients with oligoprogression was 8.7 months (95% CI 6.5–11.0). Notably, only two patients (4.5%) developed oligoprogression at the first radiologic assessment after osimertinib initiation, suggesting that the potential for early misclassification was limited. Among patients with oligoprogression, 36 patients (81.8%) received radiotherapy while continuing osimertinib, whereas six patients (13.6%) continued osimertinib without local ablative treatments and two patients (4.5%) discontinued osimertinib to receive subsequent-line therapy. Radiotherapy was the only local ablative treatment used in the cohort, and none of the patients underwent surgery for oligoprogression. Among patients treated with radiotherapy at oligoprogression (*n* = 36), 25 patients (69.4%) received a single course of radiotherapy, eight patients (22.2%) received two courses, two patients (5.6%) received three courses, and one patient (2.8%) received five courses. The median time to osimertinib discontinuation was significantly longer in patients treated with radiotherapy for oligoprogression than in those who continued osimertinib without local ablative treatments (8.5 months [95% CI 6.3–10.8] vs. 3.0 months [95% CI 2.3–3.6]; log-rank *p* = 0.001). The median OS was significantly longer in patients with oligoprogression compared to those with systemic progressive disease (57.7 months [95% CI 42.2–73.3] vs. 36.5 months [95% CI 19.4–53.7]; log-rank *p* = 0.007) (Supplementary Figure S2). Among patients who experienced progression on osimertinib monotherapy, median PPS was significantly longer in patients with oligoprogression than in those with systemic progression (28.5 months [95% CI 26.5–30.5] vs. 8.7 months [95% CI 0.0–22.0]; log-rank *p* = 0.001) (Supplementary Figure S3). Patients with oligoprogression who received radiotherapy had a significantly longer median PPS than those who did not receive radiotherapy (28.5 months [95% CI 25.4–31.6] vs. 11.1 months [95% CI not estimable]; log-rank *p* = 0.002) (Supplementary Figure S4). By the end of the data collection period, 13 patients who received radiotherapy for oligoprogression and two patients who continued osimertinib without local ablative treatments remained on osimertinib monotherapy.

**Figure 2:**
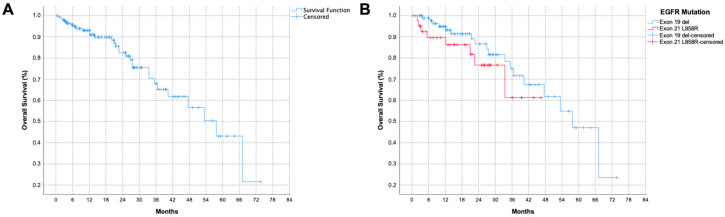
Kaplan–Meier curves for overall survival in (**A**) the overall cohort and (**B**) stratified by *EGFR* mutation subtype (exon 19 deletion vs. exon 21 L858R).

During the follow-up period, 30 patients (21.0%) died. The median OS in the overall cohort was 57.7 months (95% CI 42.4–73.1), with 12- and 24-month OS rates of 93.0% (95% CI 88.6–97.5%) and 82.5% (95% CI 74.9–90.9%) (Figure 2). In the exon 19 deletion subgroup, the 12- and 24-month OS rates were numerically higher compared to the exon 21 L858R subgroup (12-month OS rates: 94.8% vs. 89.6%; 24-month OS rates: 86.6% vs. 76.6%). However, no statistically significant difference in OS was observed between the two subgroups (log-rank *p* = 0.24).

Among patients presenting with CNS metastases at diagnosis (*n* = 51), 35 patients (68.6%) received radiotherapy to these lesions, whereas 16 patients (31.4%) did not. The intracranial ORR and DCR in patients who received cranial radiotherapy in combination with osimertinib monotherapy were 97.1% and 100%, respectively (Table 2). The corresponding rates in patients who did not receive cranial radiotherapy were 75.0% and 93.8%, respectively. During the follow-up period, intracranial progression was observed in 14 patients (27.5%) with CNS metastases at diagnosis and in 12 patients (13.0%) without CNS metastases at diagnosis (log-rank *p* = 0.011). The median time to intracranial progression was 41.4 months (95% CI 1.7–81.2) in patients with CNS metastases at diagnosis who received radiotherapy, whereas the median time was not reached in patients with CNS metastases at diagnosis who did not receive radiotherapy, nor in those without CNS metastases at diagnosis.

Of the patients in whom osimertinib was discontinued due to disease progression (*n* = 69), 47 patients (68.1%) received second-line therapy, 20 patients (29.0%) received third-line therapy, and four patients (5.8%) received fourth-line therapy. The most frequently used second-line regimen was platinum (either cisplatin or carboplatin) plus pemetrexed (*n* = 23; 48.9%), followed by platinum (either cisplatin or carboplatin) plus pemetrexed plus pembrolizumab (*n* = 3; 6.4%) and carboplatin plus paclitaxel (*n* = 3; 6.4%) (Supplementary Table S2). Histologic transformation to small cell carcinoma occurred in four patients (5.8%), and an *ALK* fusion was identified in one patient (1.4%). Among the 22 patients (31.9%) who did not receive second-line therapy, seven (10.1%) died at the time of progression or shortly thereafter, five (7.2%) were deemed unfit for further systemic treatment because of poor performance status, and ten (14.5%) were lost to follow-up after progression. Because of the substantial heterogeneity of post-progression treatment regimens and the limited number of patients within individual treatment groups, meaningful comparisons of PPS according to second-line therapy were not feasible.

Using a significance threshold of *p* < 0.1, univariable Cox regression analysis revealed associations between PFS and the following covariates: exon 21 L858R mutation (HR = 2.51 [95% CI 1.56–4.05]; *p* < 0.001), involvement of ≥3 metastatic organ systems (HR = 1.67 [95% CI 0.98–2.85]; *p* = 0.059), liver metastasis (HR= 2.10 [95% CI 1.17–3.76]; *p* = 0.013), bone metastasis (HR = 1.58 [95% CI 1.02–2.43]; *p* = 0.040), and adrenal gland metastasis (HR = 4.34 [95% CI 2.24–8.39]; *p* < 0.001) (Table 3). The multivariable Cox regression analysis demonstrated that exon 21 L858R mutation (HR = 2.07 [95% CI 1.27–3.37]; *p* = 0.003) and adrenal gland metastasis (HR = 4.41 [95% CI 2.02–9.63]; *p* < 0.001) were independently associated with worse PFS. Among patients with oligoprogression, univariable Cox regression analysis showed that receipt of radiotherapy for oligoprogression was the only variable significantly associated with time to osimertinib discontinuation following documented oligoprogression (HR = 0.16 [95% CI 0.05–0.52]; *p* = 0.002) (Supplementary Table S3).

In the safety analysis, 79 patients (55.2%) experienced any-grade osimertinib-related adverse events, while grade ≥ 3 adverse events occurred in nine patients (6.3%) (Table 4). The most frequently reported adverse events were rash (23.1%), diarrhea (16.8%), thrombocytopenia (10.5%), anemia (9.8%), and fatigue (8.4%). The most commonly observed grade ≥ 3 adverse events were rash (2.1%) and diarrhea (1.4%). Notably, one patient developed a grade 4 thromboembolic event requiring prolonged hospitalization.

## 4. Discussion

In this nationwide, multicenter, retrospective study, the real-world effectiveness and safety outcomes of first-line osimertinib monotherapy were comparable to those reported in pivotal clinical trials. Patients with exon 19 deletion achieved numerically higher response rates and experienced longer median PFS and OS than exon 21 L858R subgroup. Multivariable analysis identified exon 21 L858R mutation and adrenal gland metastasis at diagnosis as independent factors associated with worse PFS. Exploratory analyses further suggested that oligoprogression was associated with more favorable outcomes, with local ablative treatments enabling prolonged continuation of osimertinib treatment.

While the median PFS of 17.6 months was similar to that observed in the FLAURA trial and in the osimertinib monotherapy arm of the FLAURA2 trial, higher ORR (88.1%) and longer median OS (57.7 months) were observed in our cohort [7,16]. In addition to immature OS data in our cohort, several additional factors may partly account for this observation. In our cohort, a higher complete response rate was observed compared with the FLAURA trial (9.8% vs. 3%) and the osimertinib monotherapy arm of the FLAURA2 trial (9.8% vs. 1%), which may have contributed to longer survival in a subset of good responders [7,16]. The younger median age of our cohort compared with that of the FLAURA trial (59 vs. 64 years) and the osimertinib monotherapy arm of FLAURA2 (59 vs. 62 years) may have influenced the tolerability of subsequent-line treatments [7,16]. Additionally, improved second- and later-line systemic treatment options may have contributed to this observation, while the relatively high rate of local ablative treatments (81.8%) with continued osimertinib in cases of oligoprogression may also have played a role. As OS comparisons according to progression pattern are inherently susceptible to immortal time bias, we additionally evaluated PPS from the time of first documented progression. The persistence of a significant PPS advantage among patients with oligoprogression suggests that the observed survival difference is unlikely to be explained solely by immortal time bias. Nevertheless, the exploratory nature of this analysis and the potential influence of residual confounding should be acknowledged. Furthermore, the impact of individual second-line treatment strategies on PPS and OS could not be reliably assessed because of the substantial heterogeneity and limited sample sizes of the post-progression treatment groups. A recent Swiss cohort study including patients treated with first-line osimertinib (*n* = 148) reported a similar median OS of 51.6 months, with oligoprogression in 77% of patients and local ablative treatment applied in 45% of oligoprogressive cases [10]. Consistent with our findings, Schuler et al. reported significantly longer OS in patients with oligoprogression compared with those with systemic progression (51.6 vs. 26.4 months; *p* = 0.004) [10]. Another study from China (*n* = 282) reported an oligoprogression rate of 37%, with local ablative treatment applied in 46% of oligoprogressive cases, and demonstrated longer OS in patients with oligoprogression compared with those with systemic progression (35.4 vs. 24.8 months; *p* = 0.01) [11]. However, neither study demonstrated a significant OS benefit associated with local ablative treatments in the setting of oligoprogression [10,11]. Together with our findings, these results suggest that oligoprogressive disease in metastatic *EGFR*-mutant NSCLC may constitute a distinct clinical entity, highlighting the need for prospective studies evaluating tailored therapeutic strategies for this subgroup. Nevertheless, given the inherent limitations of indirect cross-trial comparisons, any comparisons with other studies should be considered hypothesis-generating rather than definitive and interpreted within the context of differences in patient populations, study methodologies, and treatment settings.

Previous studies have consistently highlighted a prognostic disparity between the two classical *EGFR* variants, with the exon 21 L858R mutation conferring a worse prognosis than the exon 19 deletion [5,6,7,16,17,18,19,20]. In the FLAURA trial, first-line osimertinib monotherapy resulted in distinct median PFS intervals of 21.4 and 14.4 months for the exon 19 deletion and exon 21 L858R cohorts, respectively [7]. This survival trend was mirrored in the osimertinib monotherapy cohort of the FLAURA2 trial, which reported median PFS intervals of 19.4 and 13.9 months for patients with exon 19 deletions and exon 21 L858R mutations, respectively [16]. In addition to the PFS benefit, significant OS differences were observed between patients with exon 19 deletion and those with exon 21 L858R mutations receiving first-line osimertinib monotherapy in both the FLAURA and FLAURA2 trials [5,6]. These findings were supported by a recent large-scale retrospective analysis of 1267 patients receiving first-line osimertinib, which reported significantly improved OS in the exon 19 deletion subgroup compared with the exon 21 L858R subgroup (adjusted HR = 1.43 [95% CI 1.22–1.67]; *p* < 0.001) [17]. The precise biological mechanisms underlying the more favorable outcomes observed in patients with exon 19 deletion remain to be fully elucidated. However, several studies have suggested that tumors harboring exon 19 deletions may exhibit greater sensitivity to *EGFR* inhibition than those harboring exon 21 L858R mutations [21]. Furthermore, differences in resistance mechanisms, co-mutation profiles, and tumor biology have been proposed as potential explanations for the consistently superior outcomes observed in patients with exon 19 deletion across multiple *EGFR*-TKI studies [22,23,24]. Collectively, these findings support the growing concept that exon 19 deletion and exon 21 L858R mutations may represent biologically distinct disease subtypes rather than interchangeable *EGFR*-mutant populations [25].

Through its ability to cross both the intact blood–brain barrier and the brain–tumor barrier, osimertinib demonstrates substantial intracranial activity, resulting in decreased CNS metastatic volume and prevention of new lesions [26,27]. Our study also included an analysis of intracranial response and progression rates among patients treated with osimertinib monotherapy. The intracranial ORR in patients who did not receive cranial radiotherapy was 75.0% in our cohort, which was numerically similar to that reported in the osimertinib monotherapy arm of the FLAURA2 trial (*n* = 38; CNS ORR, 69%) [28]. The intracranial complete response rate in our cohort was 43.8%, which was also numerically similar to the 43.3% rate reported in the osimertinib monotherapy arm of the FLAURA2 trial [28]. These findings provide additional real-world data regarding the intracranial efficacy of osimertinib monotherapy. However, they should be interpreted with caution because of the limited sample size and the descriptive nature of our analysis. Whether the addition of local treatment to osimertinib improves OS or intracranial PFS remains an area of ongoing investigation, with conflicting results reported across retrospective studies [29,30]. Despite higher ORR and DCR observed in patients with CNS metastases at diagnosis who received radiotherapy, our study did not provide definitive evidence regarding the additional benefit of cranial radiotherapy, primarily due to the small sample sizes in each subgroup and the limited number of intracranial progression events. Furthermore, baseline clinical and disease characteristics may have differed between patients who did and did not receive cranial radiotherapy, limiting the comparability of these groups.

Beyond osimertinib monotherapy, treatment intensification with the addition of platinum-based chemotherapy to osimertinib in FLAURA2 and the combination of amivantamab and a third-generation *EGFR*-TKI lazertinib in MARIPOSA showed improved oncologic outcomes, albeit at the cost of increased toxicity [5,8]. Given that a uniform treatment approach is unlikely to be suitable for all patients with metastatic *EGFR*-mutant NSCLC, therapeutic decisions should incorporate patients’ performance status, anticipated hematologic toxicity from chemotherapy, dermatologic toxicity related to amivantamab plus lazertinib, the time burden of frequent clinic visits and intravenous infusions, and individual patient preferences. While younger and fit patients without significant comorbidities may derive greater benefit from intensified strategies, osimertinib monotherapy remains an effective and well-tolerated treatment option for a substantial proportion of patients with metastatic *EGFR*-mutant NSCLC.

This study has several limitations that should be acknowledged. Despite being a nationwide study, the sample size was limited due to the lack of reimbursement for osimertinib in Türkiye during the study period. The retrospective nature of the study and the limited sample size of our cohort may have introduced selection bias and should be considered when interpreting the results, particularly those derived from subgroup analyses. The retrospective design also limited our ability to evaluate potential selection factors associated with the use of local ablative treatments, as no standardized criteria were used for patient selection and the reasons underlying these treatment decisions were not systematically recorded. Therefore, residual confounding and treatment-selection bias cannot be excluded. Other key limitations are the relatively short duration of follow-up, absence of co-mutation data, and decentralized tumor response evaluation. The lack of co-mutation data may be largely attributable to restricted access to comprehensive genomic profiling in Türkiye, primarily due to reimbursement restrictions. Consequently, molecular analyses were confined to selected driver alterations in most patients. Imaging modalities and radiologic assessments were not fully standardized across participating centers, and some response evaluations were based on PET-CT findings obtained during routine clinical practice. Therefore, response assessments may have been subject to inter-center variability and may not be directly comparable to those of prospective clinical trials using strictly protocol-defined RECIST criteria.

On the other hand, the major strength of this study is the detailed evaluation of progression patterns during first-line osimertinib monotherapy in a nationwide real-world cohort, together with the exploration of local ablative treatment strategies in patients with oligoprogression. In addition, the intracranial efficacy analysis provides complementary real-world data regarding the CNS activity of osimertinib in routine clinical practice.

## 5. Conclusions

In conclusion, osimertinib monotherapy represents an effective and generally well-tolerated first-line treatment option for patients with metastatic *EGFR*-mutant NSCLC. Oncologic outcomes appear more favorable in the exon 19 deletion subgroup compared with the exon 21 L858R subgroup. While real-world median PFS mirrored that observed in pivotal trials, the integration of local ablative treatments in carefully selected patients with oligoprogression may be associated with prolonged osimertinib treatment duration and favorable post-progression outcomes. However, prospective studies are needed to determine whether this strategy translates into improved survival outcomes. The intracranial efficacy outcomes observed in this real-world cohort were numerically similar to those reported in clinical trials.

## Figures and Tables

**Table 1:** Baseline clinicopathologic characteristics of the overall study population and *EGFR* mutation subgroups.

Variables	All Group (*n* = 143)	Exon 19 Deletion (*n* = 92)	Exon 21 L858R (*n* = 41)
Age, years, median (IQR)	59.4 (50.9–68.9)	56.9 (49.6–66.5)	65.6 (53.3–71.6)
Sex, female, *n* (%)	90 (62.9)	59 (64.1)	24 (58.5)
Smoking habit, *n* (%)			
Smoker Former smoker Non-smoker	11 (7.7) 34 (23.8) 98 (68.5)	4 (4.3) 21 (22.8) 67 (72.8)	6 (14.6) 12 (29.3) 23 (56.1)
ECOG performance status, *n* (%)			
0 1 2	60 (42.0) 67 (46.9) 16 (11.2)	39 (42.4) 41 (44.6) 12 (13.0)	16 (39.0) 21 (51.2) 4 (9.8)
Histology, *n* (%)			
Adenocarcinoma Lepidic Papillary Micropapillary Acinar Solid Mucinous Unknown Squamous cell carcinoma Adenosquamous carcinoma	143 (100.0) 20 (14.0) 11 (7.7) 10 (7.0) 27 (18.9) 25 (17.5) 3 (2.1) 47 (32.9) 0 0	92 (100.0) 13 (14.1) 8 (8.7) 7 (7.6) 14 (15.2) 15 (16.3) 3 (3.3) 32 (34.8) 0 0	41 (100.0) 6 (14.6) 1 (2.4) 3 (7.3) 9 (22.0) 10 (24.4) 0 12 (29.3) 0 0
PD-L1 TPS, *n* (%)			
<1% ≥1% 1–49% ≥50% Unknown	45 (31.5) 70 (49.0) 46 (32.2) 24 (16.8) 28 (19.6)	33 (35.9) 44 (47.8) 27 (29.3) 17 (18.5) 15 (16.3)	12 (29.3) 19 (46.3) 14 (34.1) 5 (12.2) 10 (24.4)
Metastatic presentation, *n* (%)			
De novo Recurrent	124 (86.7) 19 (13.3)	81 (88.0) 11 (12.0)	35 (85.4) 6 (14.6)
Metastatic sites, *n* (%)			
Central nervous system Liver Bone Adrenal gland Contralateral lung Pleura Pericardium	51 (35.7) 18 (12.6) 76 (53.1) 13 (9.1) 32 (22.4) 26 (18.2) 4 (2.8)	31 (33.7) 9 (9.8) 51 (55.4) 6 (6.5) 23 (25.0) 21 (22.8) 3 (3.3)	17 (41.5) 8 (19.5) 20 (48.8) 7 (17.1) 5 (12.2) 3 (7.3) 1 (2.4)
Number of metastatic sites, *n* (%)			
≤2 organ systems ≥3 organ systems	118 (82.5) 25 (17.5)	75 (81.5) 17 (18.5)	35 (85.4) 6 (14.6)
Radiotherapy at metastatic diagnosis, *n* (%)	61 (42.7)	38 (41.3)	18 (43.9)
Primary lung tumor Central nervous system Bone	6 (4.2) 35 (24.5) 29 (20.3)	3 (3.3) 21 (22.8) 20 (21.7)	2 (4.9) 13 (31.7) 8 (19.5)

ECOG, Eastern Cooperative Oncology Group; IQR, Interquartile range; TPS, Tumor proportion score.

**Table 2:** Best intracranial response and progression rates according to baseline central nervous system metastasis status and treatment with radiotherapy.

Intracranial Best Response	Patients with CNS Metastasis at Diagnosis (*n* = 51)		Patients Without CNS Metastasis at Diagnosis (*n* = 92)
	Received Radiotherapy (*n* = 35)	Not Received Radiotherapy (*n* = 16)	
Complete response (CR), *n* (%)	11 (31.4)	7 (43.8)	-
Partial response (PR), *n* (%)	23 (65.7)	5 (31.3)	-
Stable disease (SD), *n* (%)	1 (2.9)	3 (18.8)	-
Progressive disease (PD), *n* (%)	0	1 (6.3)	-
Intracranial ORR, %	97.1%	75.0%	-
Intracranial DCR, %	100.0%	93.8%	-
Intracranial progression, *n* (%)	9 (25.7)	5 (31.3)	12 (13.0)
Median time to intracranial progression, months (95% CI)	41.4 (1.7–81.2)	NR	NR

CI, Confidence interval; CNS, Central nervous system; NR, Not reached; ORR, Objective response rate (CR + PR); DCR, Disease control rate (CR + PR + SD).

**Table 3:** Univariable and multivariable Cox regression analyses for progression-free survival.

Variables	Median PFS, Months (95% CI)	Univariable Analysis		Multivariable Analysis	
		HR (95% CI)	*p* Value ^a^	HR (95% CI)	*p* Value ^a^
Age <70 ≥70	18.9 (15.6–22.1) 16.0 (11.1–21.0)	0.84 (0.48–1.46)	0.53		
Sex Female Male	18.9 (16.1–21.7) 16.3 (9.7–22.8)	1.12 (0.73–1.72)	0.61		
Smoking habit Non-smoker Smoker/Former smoker	19.4 (16.6–22.1) 16.3 (9.7–22.9)	1.32 (0.83–2.10)	0.24		
ECOG PS 0–1 2	18.9 (16.2–21.6) 14.1 (8.1–20.0)	1.24 (0.67–2.29)	0.49		
Metastatic presentation De novo Recurrent	17.4 (13.6–21.3) 26.6 (2.2–51.0)	1.35 (0.69–2.63)	0.38		
*EGFR* mutation Exon 19 deletion Exon 21 L858R	22.0 (17.2–26.8) 11.7 (6.8–16.6)	**2.51 (1.56–4.05)**	**<0.001**	**2.07 (1.27–3.37)**	**0.003**
PD-L1 TPS <1% ≥1%	19.4 (10.6–28.1) 16.3 (10.7–21.9)	1.35 (0.82–2.22)	0.24		
Number of metastatic sites ≤2 organ systems ≥3 organ systems	17.6 (14.4–20.7) 13.7 (0.7–26.7)	**1.67 (0.98–2.85)**	**0.059**	0.84 (0.41–1.75)	0.65
CNS metastases No Yes	19.4 (15.9–22.8) 14.9 (8.5–21.2)	1.19 (0.76–1.85)	0.45		
Liver metastases No Yes	19.4 (16.6–22.1) 11.4 (9.3–13.6)	**2.10 (1.17–3.76)**	**0.013**	1.76 (0.87–3.53)	0.11
Bone metastases No Yes	22.0 (14.1–29.9) 15.0 (12.2–17.8)	**1.58 (1.02–2.43)**	**0.040**	1.48 (0.93–2.37)	0.10
Adrenal gland metastases No Yes	19.4 (16.8–22.0) 6.9 (5.0–8.9)	**4.34 (2.24–8.39)**	**<0.001**	**4.41 (2.02–9.63)**	**<0.001**
Contralateral lung metastases No Yes	17.4 (13.7–21.2) 18.9 (11.9–25.8)	1.04 (0.63–1.73)	0.87		
Pleural metastases No Yes	17.0 (13.2–20.7) 24.1 (4.4–43.9)	0.62 (0.35–1.11)	0.11		

^a^ Cox proportional hazards regression; CI, Confidence interval; CNS, Central nervous system; ECOG PS, Eastern Cooperative Oncology Group performance status; HR, Hazard ratio; PFS, Progression-free survival; TPS, Tumor proportion score.

**Table 4:** Osimertinib-related adverse events.

Adverse Event	Any Grade		Grade ≥ 3	
	*n*	%	*n*	%
Any adverse event	79	55.2%	9	6.3%
Rash	33	23.1%	3	2.1%
Diarrhea	24	16.8%	2	1.4%
Thrombocytopenia	15	10.5%	0	0
Anemia	14	9.8%	2	1.4%
Fatigue	12	8.4%	0	0
Nail changes	9	6.3%	0	0
Leukopenia	8	5.6%	2	1.4%
Nausea	4	2.8%	0	0
Oral mucositis	4	2.8%	0	0
ALT or AST elevation	3	2.1%	0	0
Muscle cramp	2	1.4%	0	0
Thromboembolic event	2	1.4%	1	0.7%
Alopecia	2	1.4%	0	0
Decreased appetite	2	1.4%	0	0
Peripheral neuropathy	1	0.7%	0	0

ALT, Alanine aminotransferase; AST, Aspartate aminotransferase.

## Data Availability

The data supporting the findings of this study are available from the corresponding author upon reasonable request.

## Supplementary Materials

The following supporting information can be downloaded at https://www.mdpi.com/article/10.3390/cancers18121979/s1: Figure S1: Flow diagram of patients included in the study; Figure S2: Overall survival according to progression pattern during first-line osimertinib; Figure S3: Post-progression survival according to progression pattern following first-line osimertinib; Figure S4: Post-progression survival according to receipt of local ablative treatment among patients with oligoprogression following first-line osimertinib; Table S1: Best response rates; Table S2: Second-line treatment regimens following progression on first-line osimertinib monotherapy; Table S3: Univariable Cox regression analysis for time to osimertinib discontinuation following documented oligoprogression.

## Abbreviations

The following abbreviations are used in this manuscript:

CI	Confidence interval
CNS	Central nervous system
CR	Complete response
CTCAE	Common Terminology Criteria for Adverse Events
DCR	Disease control rate
ECOG	Eastern Cooperative Oncology Group
EGFR	Epidermal growth factor receptor
HR	Hazard ratio
IQR	Interquartile range
LAT	Local ablative treatment
NSCLC	Non-small cell lung cancer
ORR	Objective response rate
OS	Overall survival
PFS	Progression-free survival
PR	Partial response
RECIST	Response Evaluation Criteria in Solid Tumors
SD	Stable disease
TKI	Tyrosine kinase inhibitor
TPS	Tumor proportion score
TRAE	Treatment-related adverse event
