# Supplementary material for: Real-World Outcomes and Progression Patterns with First-Line Osimertinib in Patients with Advanced EGFR-Mutant Non-Small Cell Lung Cancer: A Nationwide Turkish Oncology Group Study
Source: Cancers (Basel). 2026 Jun 18;18(12):1979. doi: 10.3390/cancers18121979 (PMC13297631; doi:10.3390/cancers18121979)

Supplementary Table S1. Best response rates.

**Best response**	**All Group (n=143)**	**Exon 19 Deletion (n=92)**	**Exon 21 L858R (n=41)**	**Other *EGFR* Mutations (n=10)**
Complete response (CR), n (%)	14 (9.8)	10 (10.9)	3 (7.3)	1 (10.0)
Partial response (PR), n (%)	112 (78.3)	75 (81.5)	31 (75.6)	6 (60.0)
Stable disease (SD), n (%)	7 (4.9)	4 (4.3)	2 (4.9)	1 (10.0)
Progressive disease (PD), n (%)	10 (7.0)	3(3.3)	5 (12.2)	2 (20.0)
ORR, %	88.1%	92.4%	82.9%	70.0%
DCR, %	93.0%	96.7%	87.8%	80.0%

ORR, Objective response rate (CR+PR); DCR, Disease control rate (CR+PR+SD)

Supplementary Table S2. Second-line treatment regimens following progression on first-line osimertinib monotherapy.

**Second-line treatment regimens**	**n (%)**
Platinum (either cisplatin or carboplatin) + Pemetrexed	23 (48.9)
Platinum (either cisplatin or carboplatin) + Pemetrexed + Pembrolizumab	3 (6.4)
Carboplatin + Paclitaxel	3 (6.4)
Amivantamab + Lazertinib	2 (4.3)
Cisplatin + Gemcitabine	2 (4.3)
Docetaxel	2 (4.3)
Pemetrexed	2 (4.3)
Carboplatin + Docetaxel	1 (2.1)
Pemetrexed + Bevacizumab	1 (2.1)
Amivantamab + Pemetrexed	1 (2.1)
Erlotinib	1 (2.1)
Afatinib	1 (2.1)
Methotrexate (intrathecal)[a]	1 (2.1)
Osimertinib 160 mg[a]	1 (2.1)
Lorlatinib[b]	1 (2.1)
Cisplatin + Etoposide[c]	1 (2.1)
Carboplatin + Etoposide + Atezolizumab[c]	1 (2.1)

[a] Due to progression with leptomeningeal metastases
[b] Due to detection of *ALK* fusion at progression
[c] Due to histologic transformation to small cell carcinoma

Supplementary Table S3. Univariable Cox regression analysis for time to osimertinib discontinuation following documented oligoprogression.

Variables	Median Time to Osimertinib Discontinuation After Documented Oligoprogression, Months (95% CI)	Univariable Analysis	
		**HR (95% CI)**	***P* value[a]**
Age			
• <70	7.8 (5.2 – 10.4)	1.61 (0.63 – 4.08)	0.32
• ≥70	3.45 (3.37 – 3.53)		
Sex			
• Female	6.8 (2.8 – 10.7)	0.86 (0.38 – 2.0)	0.72
• Male	8.9 (0.1 – 17.7)		
Smoking habit			
• Non-smoker	6.2 (1.3 – 11.1)	0.72 (0.30 – 1.73)	0.46
• Smoker/Former smoker	9.0 (5.7 – 12.4)		
ECOG PS			
• 0-1	7.6 (3.8 – 11.4)	1.30 (0.39 – 4.38)	0.67
• 2	8.5 (0.4 – 16.6)		
Metastatic presentation			
• De novo	7.8 (2.8 – 12.8)	1.32 (0.45 – 3.90)	0.61
• Recurrent	6.8 (2.7 – 10.9)		
EGFR mutation			
• Exon 19 deletion	8.9 (8.1 – 9.8)	1.31 (0.58 – 2.95)	0.51
• Exon 21 L858R	6.2 (2.1 – 10.3)		
PD-L1 TPS			
• <1%	4.9 (2.0 – 7.8)	1.04 (0.44 – 2.44)	0.93
• ≥1%	7.6 (3.1 – 12.0)		
Number of metastatic sites			
• ≤2 organ systems	7.8 (4.4 – 11.2)	1.57 (0.62 – 3.95)	0.34
• ≥3 organ systems	4.5 (0.2 – 8.9)		
CNS metastases			
• No	7.8 (2.0 – 13.6)	1.46 (0.65 – 3.27)	0.36
• Yes	7.6 (2.9 – 12.3)		
Liver metastases			
• No	7.8 (5.1 – 10.5)	1.12 (0.26 – 4.83)	0.88
• Yes	4.0 (3.2 – 4.8)		
Bone metastases			
• No	6.2 (2.3 – 10.0)	1.21 (0.55 – 2.66)	0.63
• Yes	8.9 (6.0 – 11.9)		
Adrenal gland metastases			
• No	7.8 (3.2 – 12.4)	0.99 (0.33 – 2.95)	0.98
• Yes	4.5 (0.0 – 10.0)		
Contralateral lung metastases			
• No	7.6 (2.3 – 12.9)	0.67 (0.28 – 1.61)	0.37
• Yes	7.8 (0.8 – 14.8)		
Pleural metastases			
• No	8.5 (5.0 – 12.1)	2.39 (0.79 – 7.26)	0.12
• Yes	3.5 (2.6 – 4.3)		
Radiotherapy for oligoprogression			
• No	3.0 (2.3 – 3.6)	**0.16 (0.05 – 0.52)**	**0.002**
• Yes	8.5 (6.3 – 10.8)		

[a] Cox proportional hazards regression
CI, Confidence interval; CNS, Central nervous system; ECOG PS, Eastern Cooperative Oncology Group performance status; HR, Hazard ratio; TPS, Tumor proportion score

Supplementary Figure S1. Flow diagram of patients included in the study.

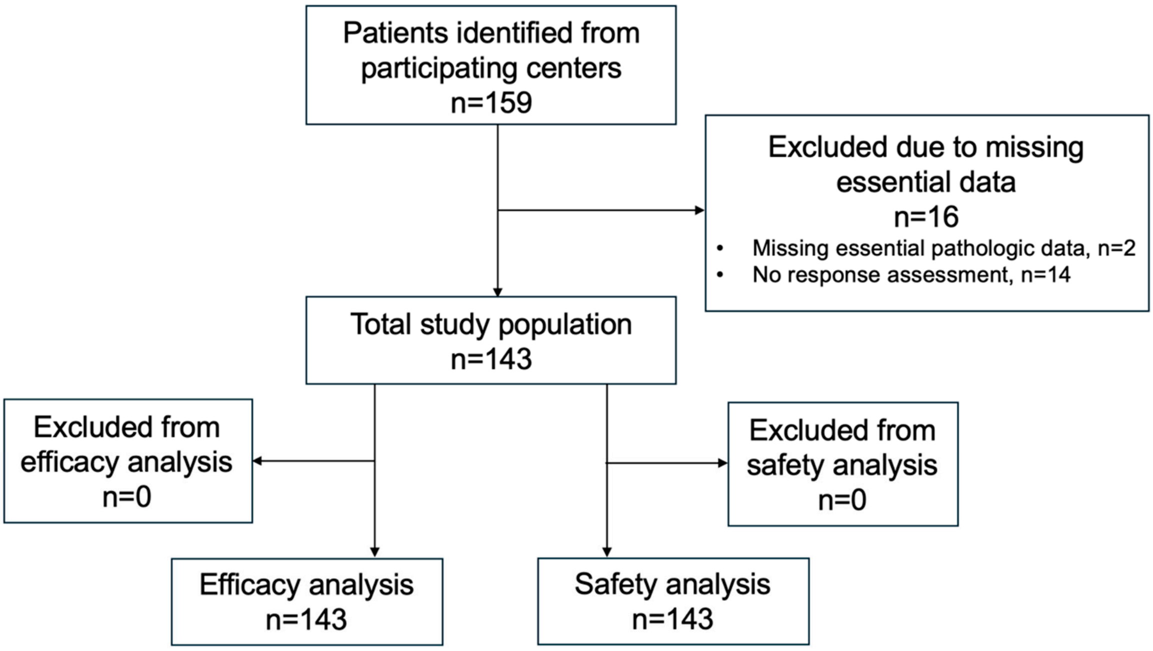

Supplementary Figure S2. Overall survival according to progression pattern during first-line osimertinib.

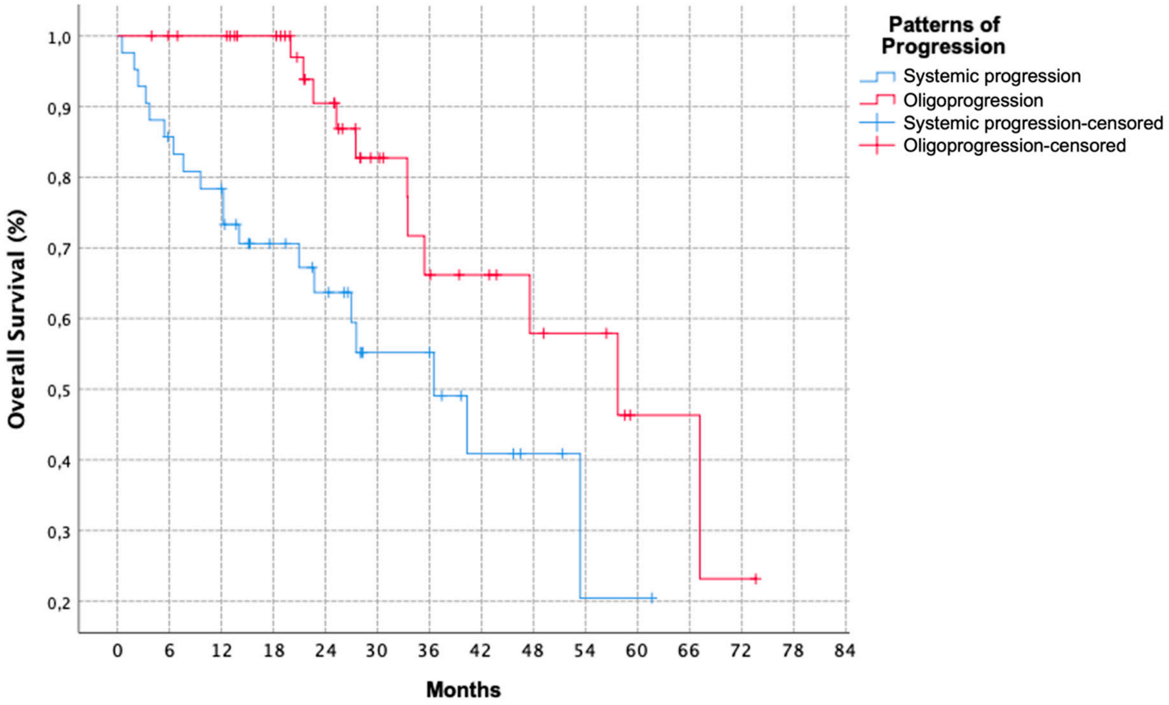

Supplementary Figure S3. Post-progression survival according to progression pattern following first-line osimertinib.

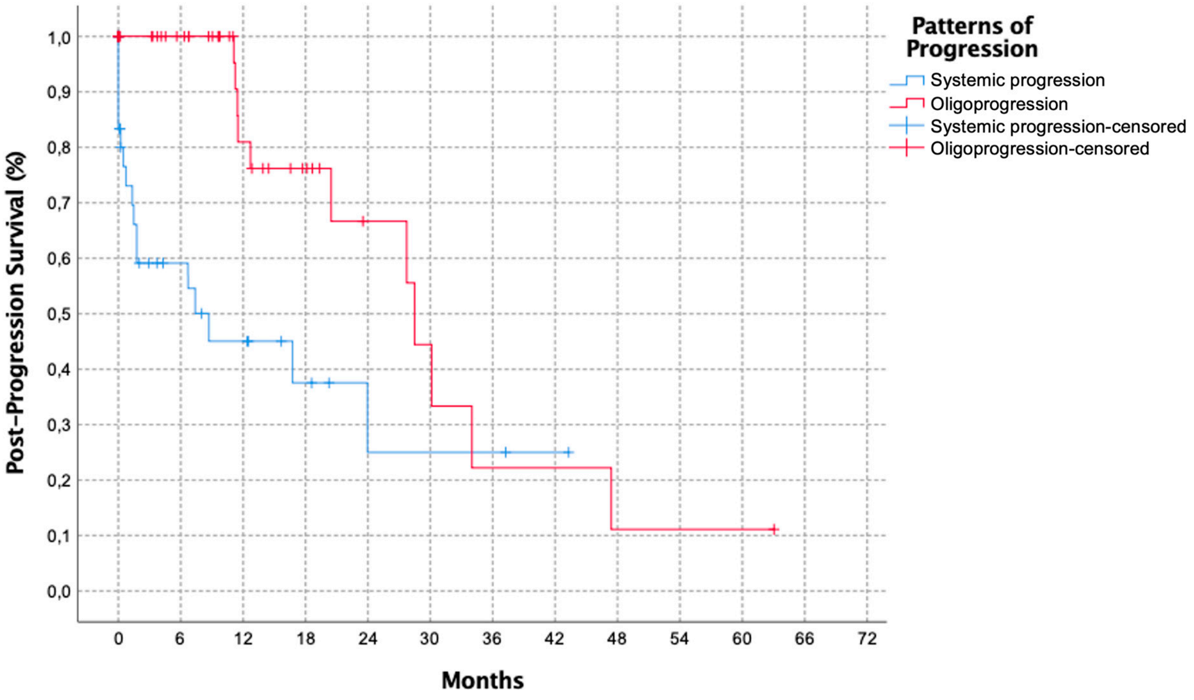

Supplementary Figure S4. Post-progression survival according to receipt of local ablative treatment among patients with oligoprogression following first-line osimertinib.

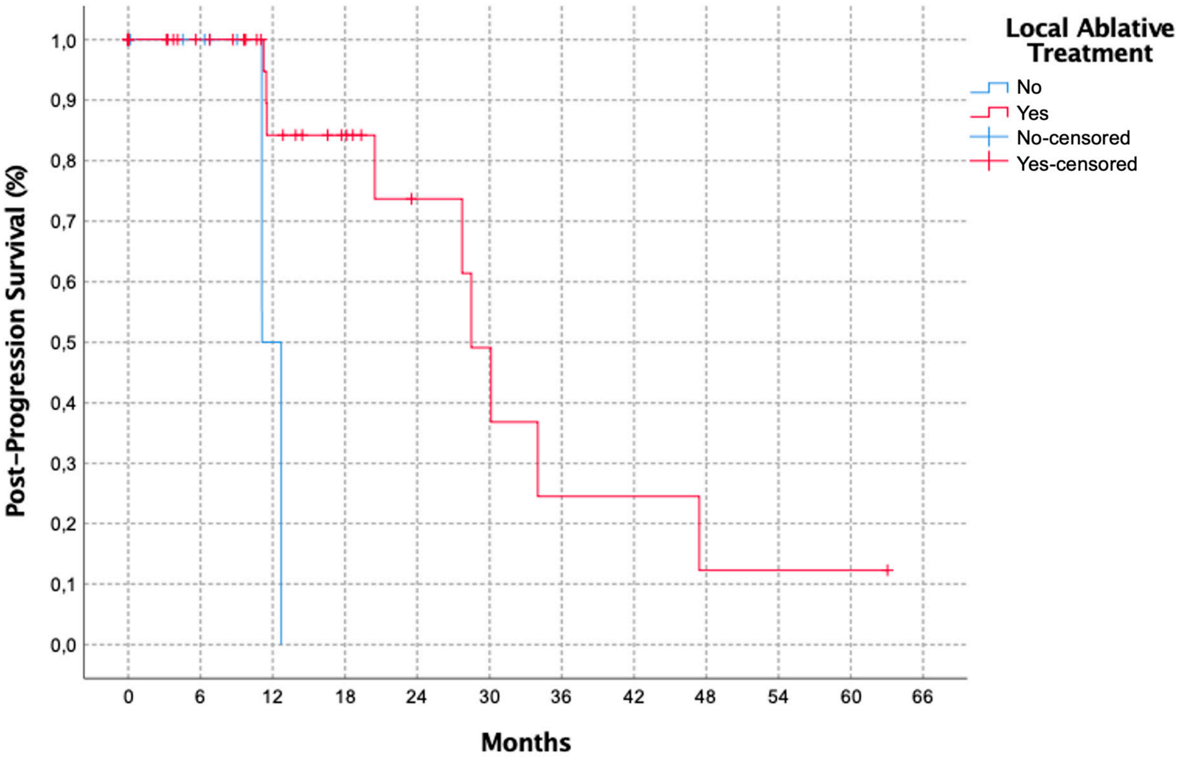

Supplement: Supplementary file 1 [file cancers-18-01979-s001.zip › cancers-4342297-supplementary.pdf]
